# Rational Design of 2D Supramolecular Networks Switchable by External Electric Fields

**DOI:** 10.1021/acsnano.3c09775

**Published:** 2024-01-23

**Authors:** Fernando P. Cometto, Nicolás Arisnabarreta, Radovan Vanta, Daniela K. Jacquelín, Vijay Vyas, Bettina V. Lotsch, Patricia A. Paredes-Olivera, E. Martín Patrito, Magalí Lingenfelder

**Affiliations:** † Max Planck-EPFL Laboratory for Molecular Nanoscience and IPHYS, EPFL, Lausanne, CH 1015, Switzerland; ‡ Instituto de Investigaciones en Fisicoquímica de Córdoba (INFIQC), CONICET, Ciudad Universitaria, Córdoba X5000HUA, Argentina; § Departamento de Fisicoquímica, Facultad de Ciencias Químicas, Universidad Nacional de Córdoba (UNC), Ciudad Universitaria, Córdoba X5000HUA, Argentina; ∥ Max Planck Institute for Solid State Research, Stuttgart D-70569, Germany; ⊥ Departamento de Química Teórica y Computacional, Facultad de Ciencias Químicas, Universidad Nacional de Córdoba (UNC), Ciudad Universitaria, Córdoba X5000HUA, Argentina; ¶ Department of Chemistry, University of Munich (LMU), Munich 81377, Germany

**Keywords:** self-assembly, scanning tunneling microscopy, external electric field, phase behavior, molecular switch, supramolecular chemistry, 2D networks

## Abstract

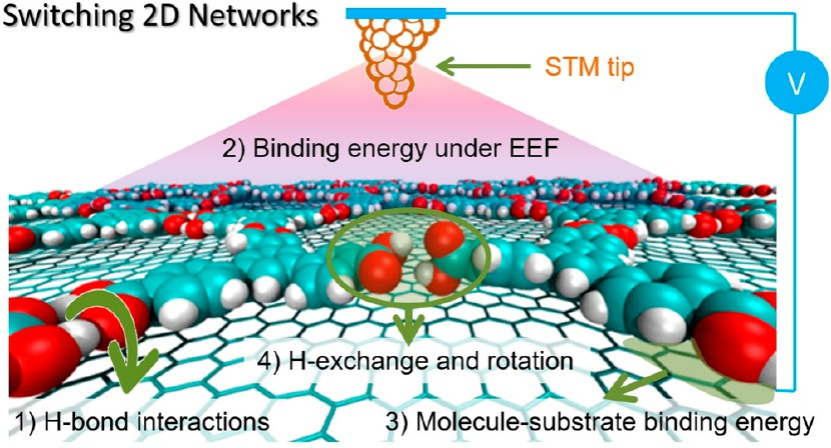

The reversible formation of hydrogen bonds is a ubiquitous mechanism for controlling molecular assembly in biological systems. However, achieving predictable reversibility in artificial two-dimensional (2D) materials remains a significant challenge. Here, we use an external electric field (EEF) at the solid/liquid interface to trigger the switching of H-bond-linked 2D networks using a scanning tunneling microscope. Assisted by density functional theory and molecular dynamics simulations, we systematically vary the molecule-to-molecule interactions, i.e., the hydrogen-bonding strength, as well as the molecule-to-substrate interactions to analyze the EEF switching effect. By tuning the building block’s hydrogen-bonding ability (carboxylic acids vs aldehydes) and substrate nature and charge (graphite, graphene/Cu, graphene/SiO_2_), we induce or freeze the switching properties and control the final polymorphic output in the 2D network. Our results indicate that the switching ability is not inherent to any particular building block but instead relies on a synergistic combination of the relative adsorbate/adsorbate and absorbate/substrate energetic contributions under surface polarization. Furthermore, we describe the dynamics of the switching mechanism based on the rotation of carboxylic groups and proton exchange, which generate the polarizable species that are influenced by the EEF. This work provides insights into the design and control of reversible molecular assembly in 2D materials, with potential applications in a wide range of fields, including sensors and electronics.

## Introduction

Molecular self-assembly is one of the most used approaches nowadays for on-surface creation of nanostructures.^1−3^ The next frontier for the fabrication of molecular nanodevices is to be able to dynamically control the structure–function properties of the nanostructures on demand, for example using external fields.^4^

**Scheme 1:**
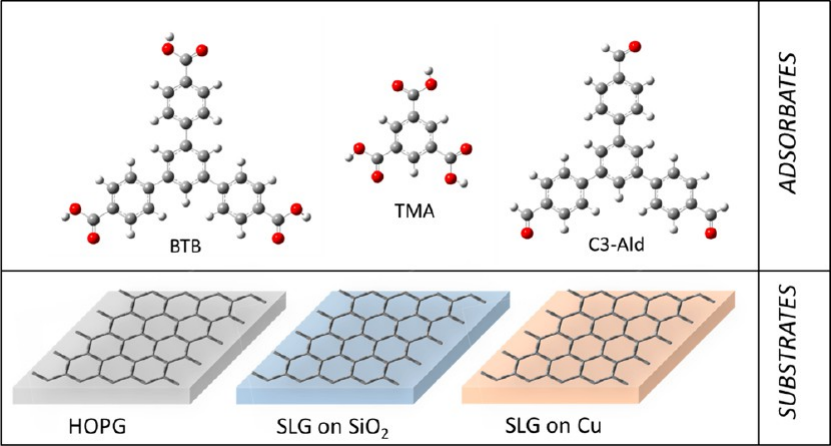
Adsorbates (BTB, TMA, and C3-Ald) and the Different Graphene-like Substrates (HOPG, SLG/SiO_2_, and SLG/Cu) Used in This Work

The ability of controlling the morphology of these architectures in an easy, fast, and predictable way is being explored for several applications in fields like sensing,^5^ catalysis,^6^ and host–guest chemistry.^7−10^ 2D supramolecular networks based on noncovalent interactions such as H-bonding build up well organized and potentially switchable engineered nanostructures.^11,12^ Several variables, such as adsorbate concentration,^13−15^ substrate symmetry, and solvent nature,^16−19^ directly impact the intermolecular bonding landscape and, thus, the final polymorphic outcome. The most popular building blocks among the H-bond-linked 2D supramolecular networks are trimesic acid (TMA)^12,16,20−23^ and its larger analogue 1,3,5-tris(4-carboxyphenyl)-benzene (BTB)^13,24−27^ (Scheme 1) normally adsorbed at the highly oriented pyrolytic graphite (HOPG)/liquid interface.

Scanning tunneling microscopy (STM) is used not only to obtain submolecular images but also to control the assembly of 2D molecular architectures by the rational employment of the external electric field (EEF) produced between the tip and the sample. Surprisingly, in a previous work we discovered that molecules with carboxylic functionalities such as BTB on HOPG undergo an immediate and fully reversible transition from an open porous nanoarchitecture at negative sample bias to close-packed polymorphs at positive sample bias at the solid/liquid interface.^13^

Since this report, plenty of efforts have been made by the community to obtain a rational understanding of the mechanism that governs the EEF-based switching phenomena at the solid/liquid interface.^8,13,20,26−30^ While the response of BTB supramolecular architectures to the EEF at the HOPG/liquid interface has been reproduced by many groups, controversies remain in the literature for the case of TMA. Some authors claim that the switching is possible but does not always take place,^27^ while others suggest that TMA adlayers are EEF-sensitive at room temperature^20^ or only above certain temperatures.^29^ Shern-Long Lee^29^ and co-workers have recently shown that TMA motifs will switch as a response to the EEF direction only in certain conditions, that is, above 45 °C at the solid/liquid interface. Conversely, they report the absence of TMA switching properties at room temperature, in contrast to the study reported by Ubink et al.^20^ It is clear that such switching properties are highly sensitive to different factors, including solvent polarity, temperature, and solvent purity (water traces).

In this article we provide a rationale to design surface supported supramolecular networks that are switchable by controlling the electric field direction between the STM tip and the sample at the solid/liquid interface. To do so, we analyzed the EEF switching effect by systematically varying both the molecule-to-molecule interactions, i.e., the H-bonding strength, and the molecule-to-substrate interactions. The H-bonding strength was varied by keeping the triaryl phenyl motif and replacing the H-bonding carboxyl groups in BTB by aldehydes in 1,3,5-tris(4-formylphenyl)benzene, C3-Ald (the synthesis of C3-Ald is described in the Supporting Information (SI)). On the other hand, the intermolecular energetics effect was also studied by removing the peripheral aryl groups around benzene in BTB to tricarboxyl-substituted benzene in TMA (Scheme 1). Furthermore, the energetics regarding molecule-to-substrate interactions were modified by employing HOPG as well as single layer graphene (SLG) supported on SiO_2_ and on a Cu surface (Scheme 1). To prove the importance of such a delicate energetic balance, we show how the BTB switching properties can be turned off by altering the molecule-to-substrate interactions via a substrate replacement.

## Results and Discussion

### Effects Contributing to the Supramolecular Switching Mechanism

#### Adsorbate/Adsorbate Energy Contribution

**Figure 1:**
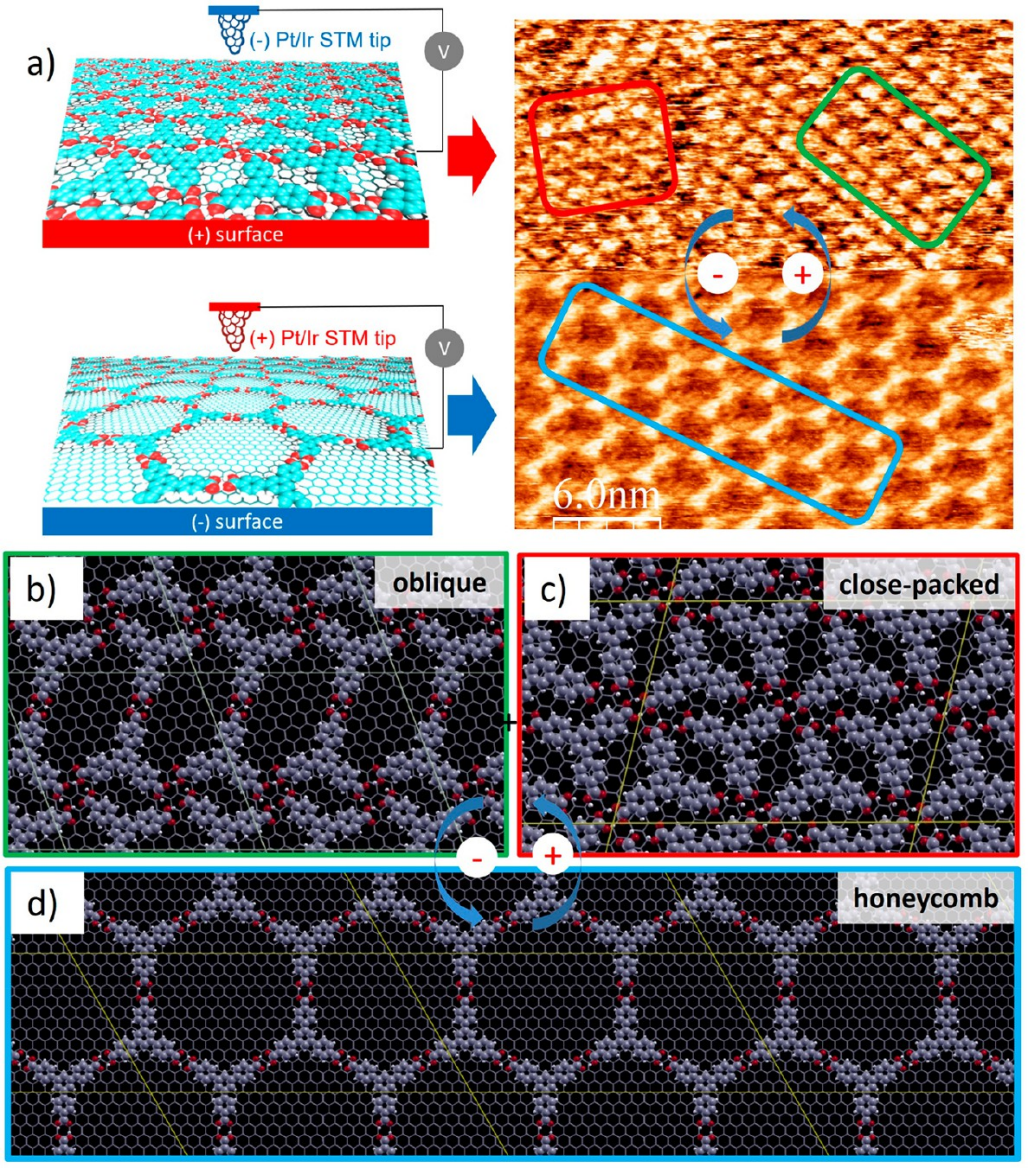
(a) Scheme (left panel) and STM image (right panel) showing the switching behavior of BTB monolayers on HOPG at the solid/liquid interface (>12.5 μM in nonanoic acid) by changing the bias voltage (V_bias_ = from −850 to +850 mV; I = 150 pA). (b–d) Equilibrium structures obtained by DFT calculations of the (b) oblique, (c) close-packed, and (d) honeycomb structures.

Typical STM images showing the bias voltage-dependent BTB polymorphisms at the HOPG/NA interface are presented in Figure 1a. At negative sample bias, only a porous honeycomb network is observed, whereas two higher molecular density structures, namely oblique and close-packed, are imaged when reversing the sample bias to positive values.^13^^13^ This switching occurs instantly when reversing the sample bias polarity, and it only affects the local area scanned under the STM tip.^13^ Global switching of BTB has been previously reported by Lackinger and co-workers^24^ by employing heat as stimulus, which affects the whole surface, in contrast to the STM electric field, which is only generated locally. This switching behavior does not show any concentration dependence and can be observed employing different organic solvents such as *n*-heptanoic acid, *n*-octanoic acid, *n*-nonanoic acid, and phenyl octane. Only at very low concentrations (2.5% of a saturated solution = 12.5 μM) the honeycomb is also imaged at positive bias, however, only at early stages of the molecular drop casting. After some minutes, the chickenwire motif evolves to the thermodynamically most stable structure at positive biases, indicating that the open architecture is only kinetically favored at positive biases. Periodic vdW-DFT calculations on the adsorption of BTB units on a graphene layer (Figure 1b–d) show the intermolecular H-bonding landscape of each of the 3 polymorphs along with their respective unit cells in total accordance with experimental studies (Figure 1a). The superstructure unit cells in Figures 1b–d present areas of 17.83, 9.28, and 13.51 nm^2^ for the honeycomb, oblique, and close-packed structural motifs, yielding BTB molecular densities of 0.22, 0.43, and 0.59 molecules/nm^2^, respectively, in agreement with STM observations. Additionally, periodic vdW-DFT calculations allow us to obtain the formation energy (E_F_) for each structure, which can be computed from the following equation:
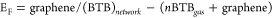
1where *n* isolated BTB molecules in the gas phase are adsorbed on the graphene surface with a given network structure. E_F_ presents two main contributions, namely, (a) intermolecular BTB–BTB interactions and (b) BTB–graphene surface interactions, as designated by the adsorption energy, E_ads_. For the honeycomb, oblique, and close-packed structures, we obtained the following formation energies per BTB molecule (E_F_/*n*): −45.5 kcal/mol, −39.3 kcal/mol, and −38.5 kcal/mol, respectively. Assuming that the average adsorption energy per BTB molecule is the same for all structures, we can attribute the differences in the formation energies to the different BTB–BTB interactions within each network. As expected, the highest interaction is observed for the honeycomb motif (Figure 1d) due to the strong hydrogen bonding formed by the head-to-head carboxylic dimers, for which we obtained a value of 16.5 kcal/mol. On the other hand, the oblique structure (Figure 1b) is built-up by only 1 head-to-head dimeric H-bond as well as 2 tetrameric cooperative H-bonds per BTB unit, whereas the close-packed phase (Figure 1c) presents highly complex and less energetic cooperative H-bonds.

**Figure 2:**
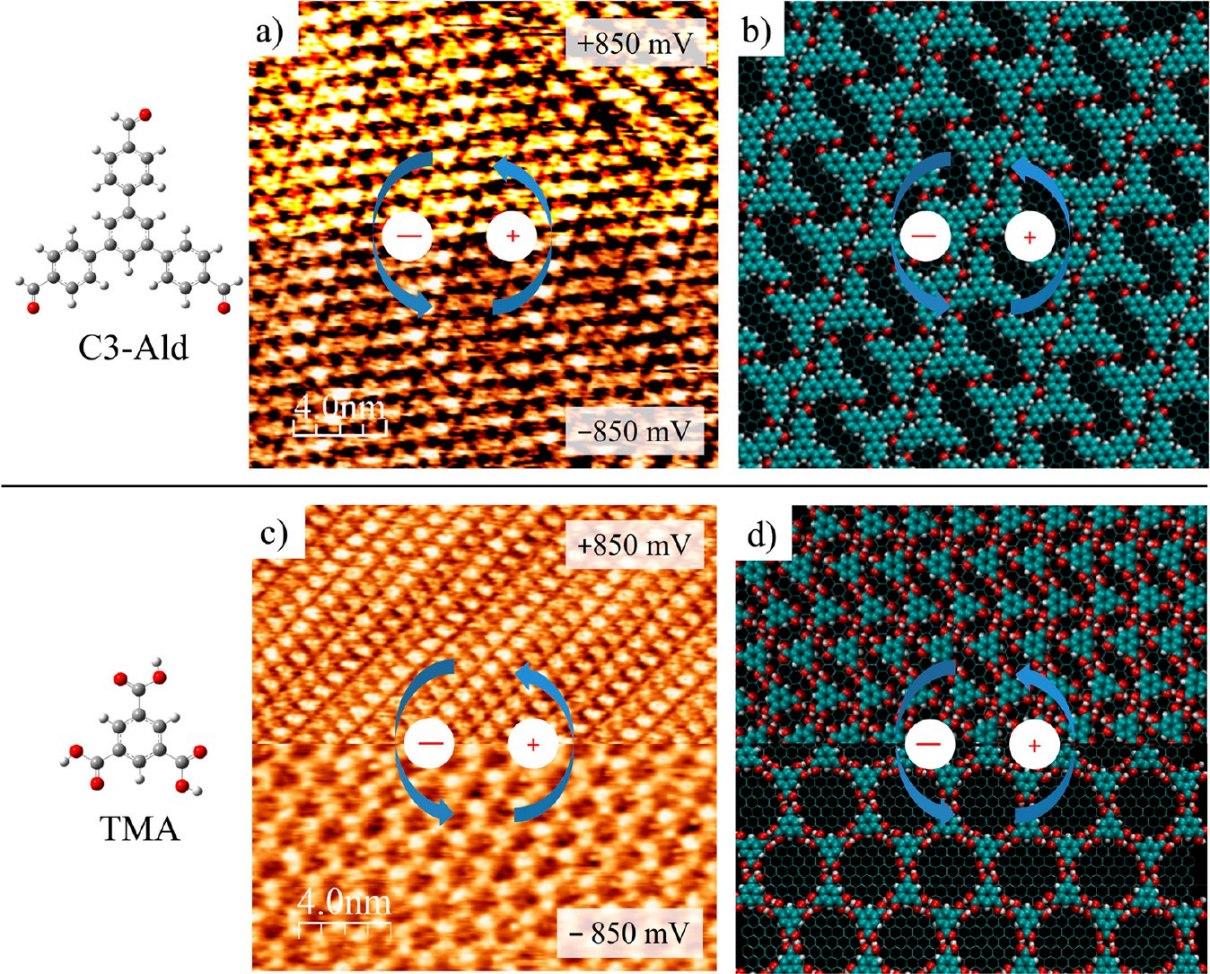
(a and c) STM images showing the typical structure of C3-Ald (a) and TMA (c) monolayers on HOPG at the solid/liquid interface obtained at different bias voltages (V_bias_ = from 850 to −850 mV; I = 150 pA). (b and d) Optimized equilibrium C3-Ald (b) and TMA (d) structures obtained by MD simulations.

To focus on the effect of the intermolecular energetics on the switching mechanism, we performed experiments with different BTB-analogue molecules, namely, TMA and C3-Ald. As observed in Scheme 1 as well as in Figure 2, C3-Ald presents a BTB backbone but with aldehyde groups instead of carboxylic acids, whereas TMA presents the same functional groups as BTB but without the peripheral phenyl rings. Figures 2a and 2c (and Figure S1) show the typical STM images obtained for C3-Ald and TMA at different potential biases after deposition of their diluted (5%) solutions at the NA/HOPG interface, respectively. Interestingly, C3-Ald builds-up a nonporous densely packed nanoarchitecture regardless of the sample bias (Figure 2a), in total contrast to its -COOH-functionalized analogue, BTB. This can be explained due to the energetics involved in the intermolecular interactions of C3-Ald motifs. While head-to-head H-bonds are highly energetic and directional, as in the case of -COOH-based structures (BTB), the interaction is much weaker in the case of -CHO-based H-bonding. This prevents the formation of H-bonded low-density porous networks. MD simulations show the formation of a stable closed packed C3-Ald structure on graphene, as observed in Figure 2b. Thus, the BTB functional group replacement (COOH → CHO) to get C3-Ald inhibits the possibility of the porous network formation on HOPG and, therefore, removes the switching behavior at the solid/liquid interface.

In contrast to C3-ald, TMA structures undergo a phase transition from the honeycomb structure (at negative bias) into a dense close-packed structure (at positive bias), as in the case of BTB. High resolution STM images showing such transitions as well as the vdW-DFT optimized structures can be observed in Figures 2c and 2d, respectively. Analyzing the TMA structure H-bonding landscape, it can be observed that, as in the case of BTB, the negative potential values favor the honeycomb structure, where each TMA molecule is interacting by 3 head-to-head H-bonds. At positive values, a similar BTB oblique structure is observed in which each TMA molecule interacts with only 1 head-to-head and 2 cooperative H-bonds. As it was noted by Velpula et al.,^27^ the structural transitions in the TMA network do not always take place upon changing the polarity of the substrate bias. Also, after several measurements, we hardly observed the TMA transition noticed by Ubink et al.,^20^ where the honeycomb structure switches into the more densely packed “*flower*” structure.

#### Surface Polarization Energy Contribution

**Figure 3:**
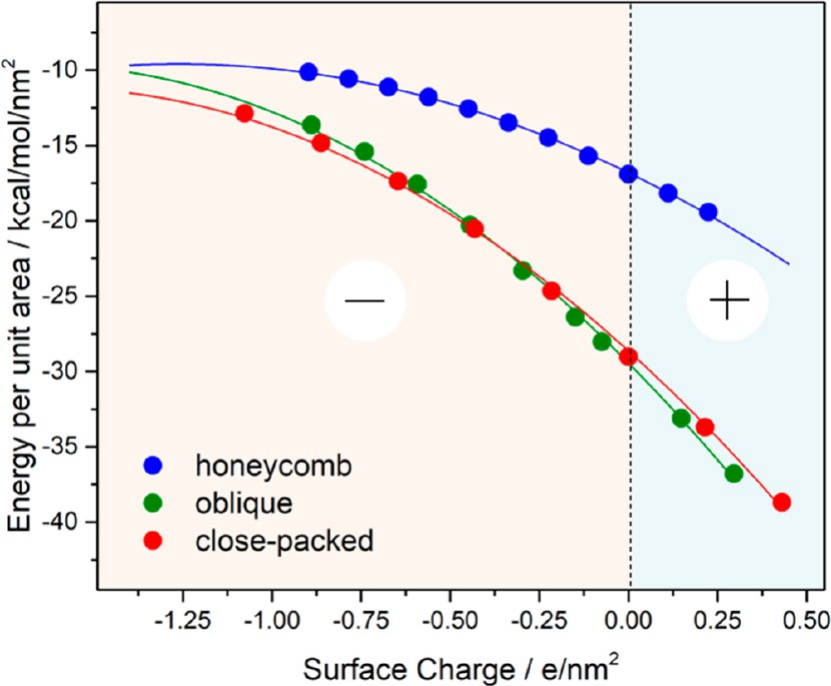
Formation energy per unit area as a function of the surface charge for the honeycomb (blue curve), oblique (green curve), and highly close-packed structures (red curve).

The switching effect between different structures implies a continuous ligand exchange between molecules from/to the solution and, consequently, involves both adsorption and desorption energies. In this context, the switching effect can also be globally interpreted in terms of the surface binding energy and the implicit related contributions therein. In agreement with the known basic concepts of chemical surface bonding, polarization of the substrate/adsorbate (induced by the electric field) strengthens the surface bond, whereas Pauli repulsion between substrate–adsorbate charge densities destabilizes the interaction. We evaluated the effect of surface polarization on the stability of the BTB structures shown in Figures 1b–d by means of vdW-DFT calculations simulating the EEF by adding (or removing) a given number of electrons from the surface. The formation energy was computed according to eq 1 and normalized by the unit cell area of each structure. Figure 3 shows the results for the calculated formation energy for different BTB structures as a function of the surface charge. As we explain next, the scenario varies depending on the surface charge:

At a surface charge equal to zero (zero surface charge), the honeycomb structure (Figure 1d) presents the less favorable formation energy (−16.9 kcal mol^–1^ nm^–2^, blue curve on Figure 3). This was expected considering the low molecular surface density (0.224 molecules/nm^2^) present in this polymorph. However, although the oblique and close-packed (Figure 1b, c, respectively) motifs present different BTB surface densities (0.431 and 0.592 molecules/nm^2^, respectively), their formation energies (−29.0 kcal mol^–1^ nm^–2^ and −28.8 kcal mol^–1^ nm^–2^, respectively) are comparable at zero surface charge. This suggests that the closed-packed H-bond network presents a lower contribution to the formation energy than that of the oblique phase, which has energetic head-to-head dimeric hydrogen bonding between opposed carboxylic groups. In other words, to present similar formation energies, the oblique phase compensates for the lower energetics arising from lower molecular surface density via formation of more energetic head-to-head H-bonds.

Outside of zero surface charge, Figure 3 shows the increase in the formation energy (|ΔE_F_|) for positively charged surfaces, meaning that reaction 1 becomes more exothermic. As observed in red and green curves in Figure 3, this stabilization is more evident for dense structures; that is, oblique and close-packed phases stabilize by 6 kcal mol^–1^ whereas the honeycomb structure is stabilized by only 3 kcal mol^–1^ when 0.25 e/nm^2^ is extracted from the system. On the contrary, injecting electrons to the graphene surface, simulating a negative sample bias, induces a destabilization of all structures due to the weakening of the BTB–graphene surface bonding. This is expected to facilitate the BTB surface diffusion, consistent with a BTB local dilution, in order to optimize molecule-to-molecule energetic H-bond interactions. Additionally, the BTB adsorption/desorption processes are also coupled to desorption/adsorption processes of solvent molecules. In this context, the binding energy of BTB molecules is more sensitive to the surface charge than the binding energy of nonanoic acid solvent molecules (see Figure S2). Therefore, when the BTB binding energy increases at positive bias, the energy of the system will be further decreased when adsorbed solvent molecules (in the pores of the honeycomb motif) are replaced by BTB units, giving rise to the high coverage polymorphs. Notably, the effect of the negative surface charge has a much higher destabilizing effect on the high coverage structures, resulting in the convergence of all curves in Figure 3 at negative surface charges. This convergence suggests that the interchange between the closed packed to open phase should occur, at negative biases. In addition, considering that solvent molecules are coadsorbed within the network pores (see Figure S3), the honeycomb binding energy vs surface charge profile (blue line in Figure 3) would present an extra stabilization and the curves’ convergence should take place at surface charges closer to zero charge. Moreover, the two high coverage structures (oblique and close-packed) present similar formation energies. Thus, it is right to predict that the interchange of the honeycomb architecture with either of these two high coverage structures when restoring the positive bias should be equally likely, as shown experimentally in Figure 1a.

#### Adsorbate/Substrate Energy Contribution

In this section we show how the surface-to-molecules interactions also play a key role in the switching process of BTB at the solid/liquid interface. Thus, to tune the adsorption energy, we used a single layer of graphene (SLG) supported on SiO_2_ and on polycrystalline Cu. STM images taken under bias conditions that allow us to visualize the HOPG lattice on Cu and SiO_2_ indicate that the HOPG surface is stable after the switching events (Figures S4).

**Figure 4:**
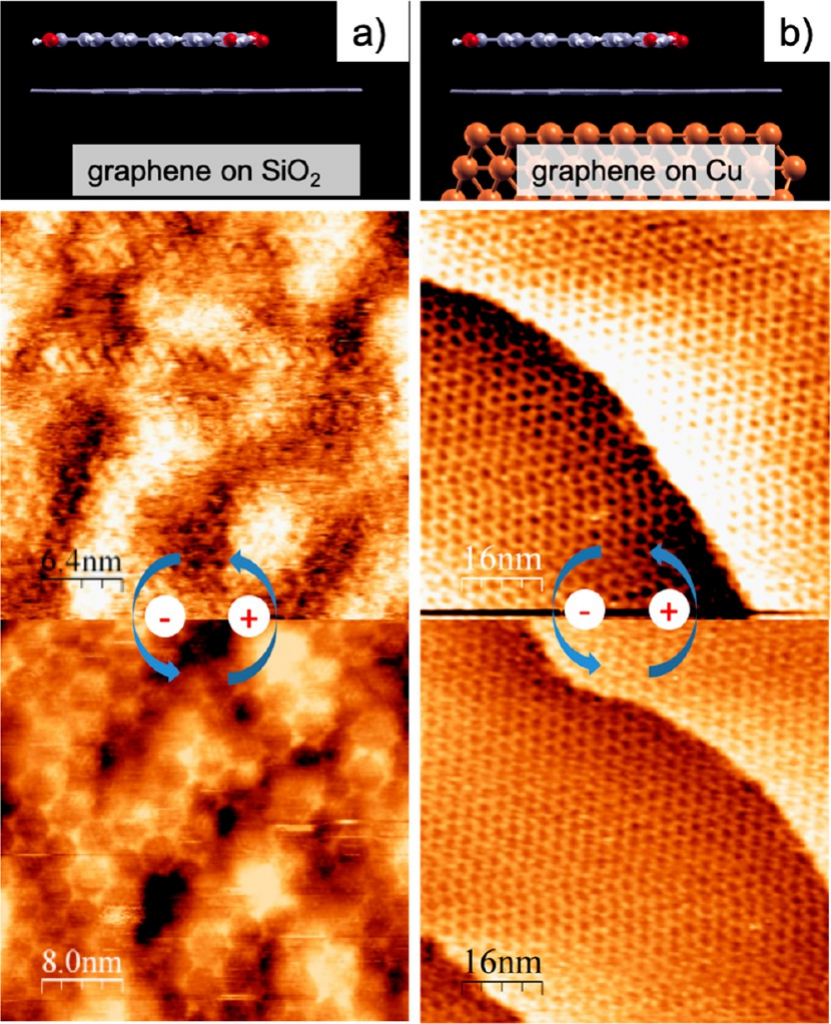
STM images showing the effect of the substrate in the voltage-induced phase transformation of BTB monolayers on SLG supported on (a) SiO_2_ and (b) Cu at the solid/liquid interface. Bias voltage (V_bias_) = from −850 to +850 mV; I = 150 pA.

For the sake of comparison, these experiments were carried out under the same conditions used for the adsorption of BTB on HOPG. Figure 4a (and Figure S4) shows STM images of BTB networks on an SLG/SiO_2_ substrate at both negative and positive sample bias. Due to the roughness of the underlying silicon oxide surface, these STM images do not present optimal smoothness as the ones obtained on flat HOPG surfaces. Nevertheless, BTB molecules can be imaged thanks to the presence of the pending SLG placed on top of SiO_2_. As observed in Figure 4a, the adsorption of BTB on SLG deposited on the SiO_2_ surface does not modify the switching behavior of BTB, since the same bias dependent polymorphism transition is obtained: densely packed (honeycomb) at positive (negative) potential values, as was previously described for BTB on HOPG.

With the aim of testing the proposed adsorption energy effect on the phase transition, BTB molecules were also absorbed on an SLG placed on a polycrystalline Cu film. In contrast to the SiO_2_ surface, the strong coupling of the graphene layer with the Cu underneath can modify the BTB/substrate bonding strength and, consequently, affect the switching behavior. Accordingly, as depicted in Figure 4b, the porous honeycomb BTB structure is always observed at the interface regardless of the polarity of the sample. Thus, we found that the BTB EEF switching effect can be switched off by changing the substrate, which is a key observation to develop a rational understanding of EEF-triggered systems at the solid/liquid interface.

**Figure 5:**
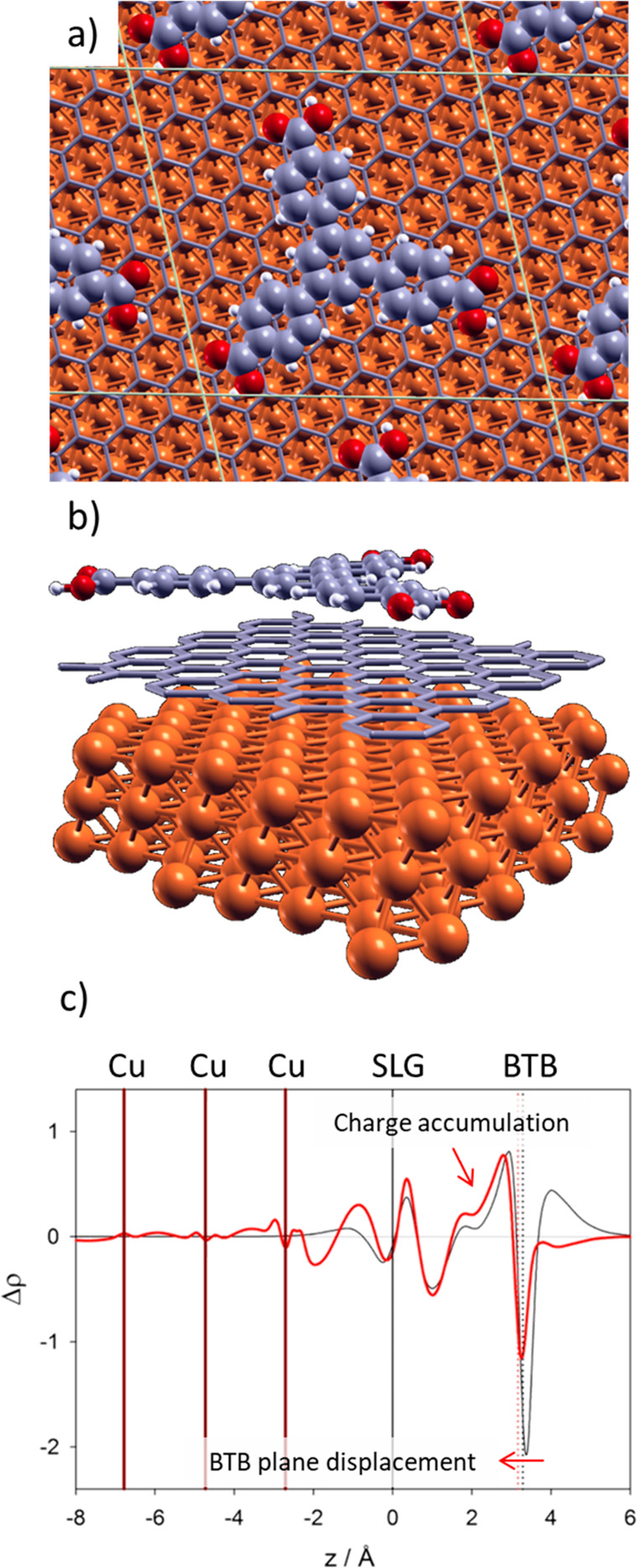
(a and b) Top view (a) and side view (b) of the equilibrium structure of BTB molecule adsorbed on SLG/Cu(111). A large unit cell was used to avoid interactions among adjacent molecules. (c) Plane-averaged charge density difference Δρ(*z*) as a function of the vertical direction *z* after BTB adsorption on SLG (black curve) and on SLG/Cu(111) (red curve). The vertical solid lines indicate the positions of the Cu and SLG planes. The vertical dotted lines indicate the position of the BTB molecule on each surface.

To understand this effect, we performed comparative DFT calculations on the adsorption of BTB on an SLG as well as on an SLG supported on the (111) Cu face. In this last case, a *top-fcc* stacking was employed (Figure 5), which is considered to be the most stable configuration. Interestingly, the results show that the binding energy (defined as the ΔE for the adsorption of BTB initially in the gas phase) on SLG/Cu(111) is −43.1 kcal/mol, whereas it is only −33.1 kcal/mol on SLG. This means that the presence of a Cu surface underneath the graphene layer strengthens the interaction by 10.0 kcal/mol. This effect is also evidenced by the shortening of the average BTB surface height, which decreases from 3.29 Å on SLG to 3.16 Å on SLG/Cu(111). The increased reactivity of graphene on Cu(111) can be understood considering that Cu induces a large polarization in the graphene electron density producing a depletion of charge above the graphene plane.^31^

The BTB/substrate bonding strengthening is evidenced in the electron density difference Δρ plots, in which the substrate and adsorbate electron densities are subtracted from the electron density of the adsorbate–substrate system. In Figure 5c we compare the plane-averaged charge density difference plots for BTB on SLG and on SLG/Cu(111). They are obtained by integrating Δρ(*x*,*y*,*z*) over the *x* and *y* coordinates to obtain Δρ(*z*) where the regions of charge accumulation and depletion are clearly observed. Above the BTB molecule, charge is depleted, while in the interfacial region between the molecule and graphene, charge is accumulated. In the case of BTB/SLG (black curve Figure 5c), the effect of graphene is to polarize the BTB electron density; however, there is no major accumulation of electron charge in the interfacial region. On the other hand, for the BTB/SLG/Cu(111) system (red curve Figure 5c), the regions of charge accumulation are more prominent in the interfacial region and integration of Δρ(*z*) in this region yields a net charge accumulation of 0.2 e. Because of the charge depletion induced by the Cu substrate, the region above graphene is positively charged. Thus, the binding energy of BTB is increased by 10.0 kcal mol^–1^. This increment in the binding energy seems to be enough to freeze the kinetically favored honeycomb structure, removing the switching behavior described for BTB in HOPG (or SLG/SiO_2_). As we have previously described for the case of diluted BTB solutions on HOPG, the honeycomb structure is first found with positive bias but, then, evolves to the close-packed structure in a few minutes.^13^ In contrast, due to the increment in the binding energy, the system is always trapped in the honeycomb structure even at positive sample biases when BTB is adsorbed on the SLG/Cu substrate.

#### Dynamics of the Switching Mechanism: Carboxyl Group Rotation and Proton Exchange

Besides the energetic considerations of the previous sections, it is also important to consider the dynamics of BTB molecules within a network. It has been hypothesized^13,27,28^ that the carboxyl groups’ deprotonation (favored under positive surface polarization) may be the driving force of the phase transition at the solid/liquid interface. Along this axis, Saeed et al.^28^ showed that the presence of water traces at the liquid/solid interface promotes the TMA phase transition at room temperature as a response to the EEF; the same effect was observed to scale with the polarity of the solvent.^21−23^ However, these events can also be rationalized as a consequence of the change in the dielectric constant of the electrical double layer, which will influence the dynamics of polarizable species. Unfortunately, the protonation state of a monolayer adsorbed on a surface at the solid/liquid interface cannot be precisely measured. Hypotheses concerning deprotonation have been taken based on simulations,^32^ as well as experiments performed under UHV, where deprotonation and recombination to H_2_ happen at around 420 K,^25,33,34^ conditions that are far away from the ones used at the solid/liquid interface. To investigate these considerations, we performed reactive molecular dynamics simulations and discovered two dynamic processes occurring within a BTB open network that do not imply permanent deprotonation, namely: (a) the H-bonding dynamic exchange process and (b) carboxylic group rotation in BTB carboxylic dimers. Accordingly, we will show that these two scenarios create polarizable species (see SI Video) which, influenced by the strong and localized EEF present locally during STM measurement, can induce molecular switching.

##### H-Bonding Dynamic Proton Exchange

**Figure 6:**
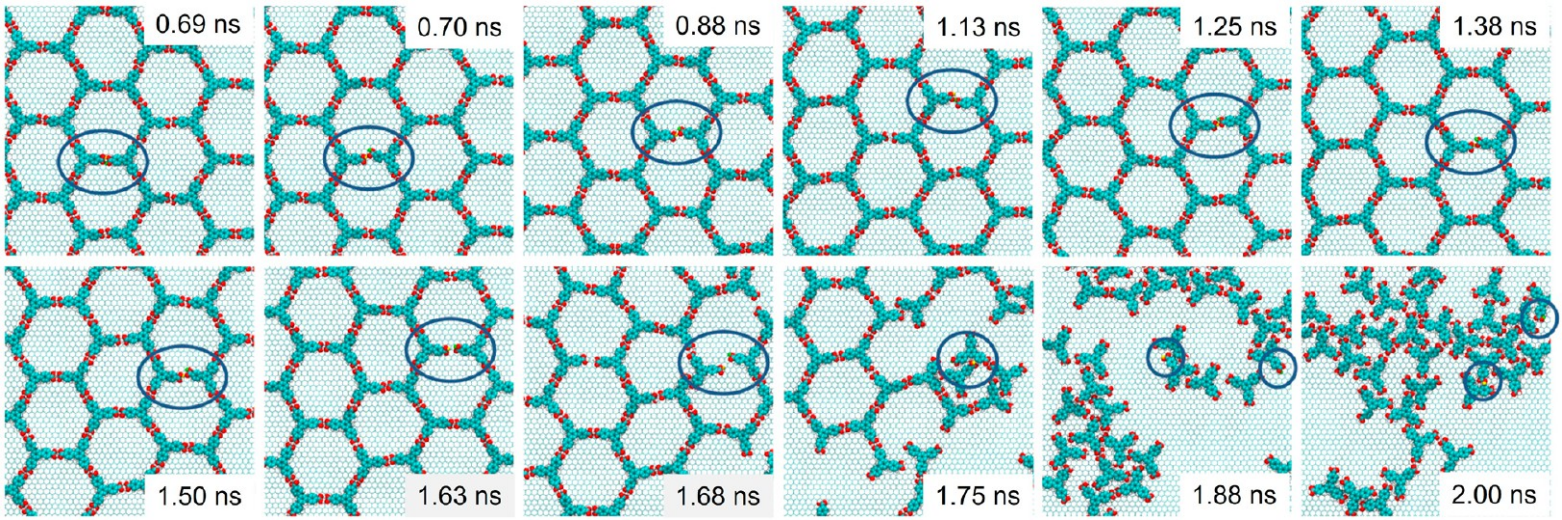
Snapshots of reactive molecular dynamics simulations showing the evolution of a BTB honeycomb structure during a temperature ramp from 350 to 650 K within 2.0 ns.

Figure 6 shows representative snapshots of the network evolution from the intact BTB honeycomb structure at 350 K to a disordered one as the temperature is raised to 650 K. The temperature ramp was applied during 2.5 ns in order to accelerate the dynamics of the system. It is important to point out that this is a statistical description, meaning the same transitions could happen at longer times and lower temperatures (smoother T ramp). Results show that at early stages, the BTB structure remains almost intact. As the dynamics proceeds, some defects in head-to-head H-bonds appear (as circled in Figure 6), promoting disorder in the adlayer until the honeycomb structure collapses.

**Figure 7:**
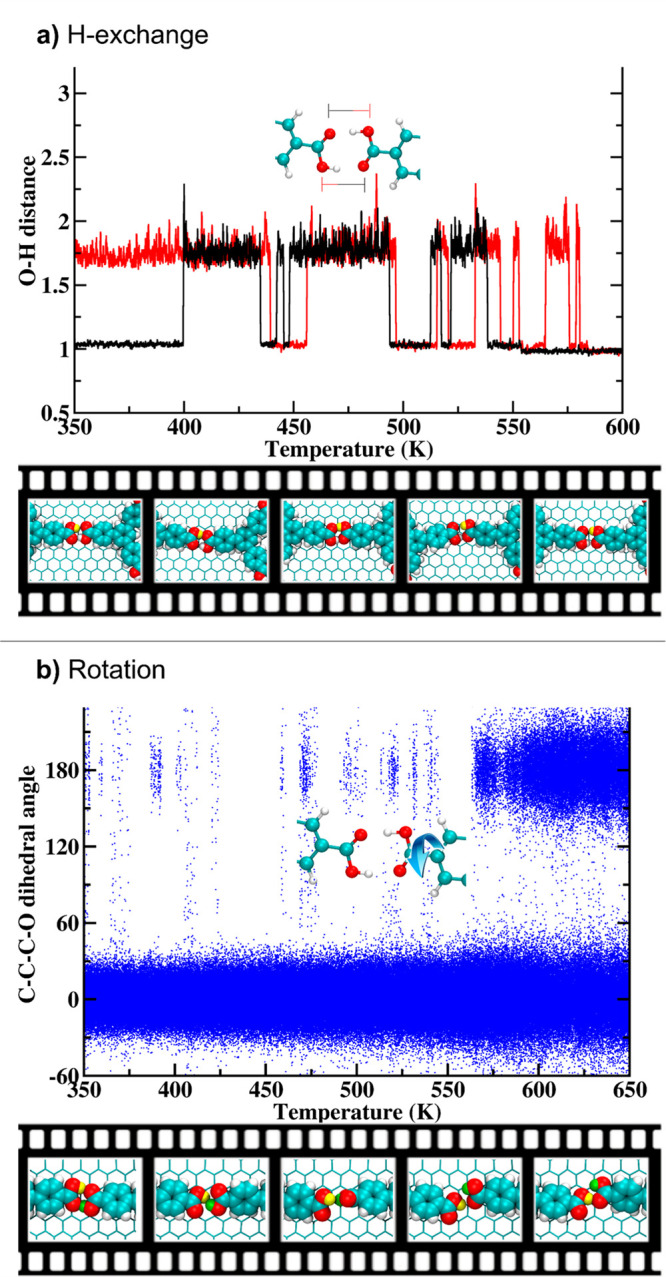
(a) Description of the H-exchange by means of O–H bond length (red profile) and H-bond length (black profile) for the head-to-head carboxylic groups in a honeycomb structure as a function of temperature. (b) Statistical description of the carboxylic group’s rotation by means of the variation of C–C–C–O dihedral angles (in degrees) for 20 BTB molecules during the temperature ramp from 350 to 650 K for BTB molecules in the honeycomb structure.

Focusing on the atomistic processes, several H-exchange processes between dimeric -COOH groups as well as rotation of carboxylic groups occur even at the beginning of the reactive dynamics (Figures 7a–b). Since the occurrence of proton exchange between adjacent carboxylic groups at all temperatures in the MD simulations indicates that this process has a low energy barrier, to support this observation, we conducted a nudge elastic band calculation^35^ (Figure S6), obtaining an energy barrier for proton exchange of 0.47 eV, which aligns with the observed proton exchange in the MD simulations. Figure 7a shows the statistical description of the proton transfer process between adjacent carboxylic groups by following the OH distances for both O atoms (see inset) during the temperature ramp. The OH distances oscillate between ∼1.0 Å (O–H covalent bond distance) and ∼1.6 Å (H-bond), showing H-exchanges in the honeycomb BTB structure at temperatures lower than 400 K in the MD simulation. When the black and red profiles overlap—at around 1.6 Å (Figure 7a), a deprotonated carboxylate group (-COO)^−^ faces a -C(OH)(OH)^+^ group. Such configurations confer a dipole moment to BTB molecules that can interact with the applied EEF between the STM tip and the sample and result in an on-surface phase transition.

##### Carboxyl Group’s Rotation

We further explored the rotation of the carboxylic groups to determine the origin of the H-bond breakage during switching. Figure 7b shows the statistical occurrence of the carboxylic group rotation during simulations by computing the C–C–C–O dihedral angles of several BTB molecules. When the entire BTB molecule lies flat on the surface (meaning null dipole moment), the C–C–C–O angle is 0.0°, and when the carboxylic group rotates around the C–COOH bond (creating a clear dipole moment), such an angle becomes 180.0°. This latter case would mean the complete breakage of the intermolecular H-bond, destabilizing the overall system. As observed in Figure 7, the occurrence of such nonzero dihedral angles increases as the dynamic proceeds. Even though only a few carboxyl rotations are observed at the beginning of the temperature ramp (early stages), they need to be taken into consideration, since the polarized species could easily interact with the highly localized and strong EEF applied during STM measurements. Furthermore, as expected, free rotations occur at high temperatures (above 550 K) where dihedral angles of 180° are observed coexisting with angle values around zero degrees.

Summarizing, after the rotation of the -COOH group that is concomitant with the H-exchange process, several H-bonds are broken, resulting in the whole BTB structure destabilization and collapse (Figure 7). Accordingly, this effect will also be enhanced by the large electric field between the STM tip and sample, contributing to the overall energetic balance of the switching process. These simulations allow us to correlate the switching behavior with the process of H-exchange and -COOH group rotation, in a reversible manner and without the need of deprotonation.

As a comprehensive discussion of the proposed switching mechanism, we initially examined the various energetic contributions. As depicted in Figure 3, a charged substrate, emulating the surface polarization due to an applied perpendicular electric field, either stabilizes or destabilizes different networks, elucidating the energetic preference for opened structures on negative surfaces and close-packed structures on positive ones. Furthermore, through reactive molecular dynamic simulations, we demonstrated that both processes (the rotation of carboxylic groups and proton transfer/exchange) can occur in stable structures (Figures 6 and 7). These processes generate a nonzero dipole moment that, when interacting with the electric field, triggers the collapse of the structure. Additionally, employing DFT calculations of BTB molecules in a vacuum, we determined the dipole moment of BTB molecules in various configurations (planar before H-exchange and/or rotation, as observed in honeycomb structures) and nonplanar asymmetric arrangements where carboxylic rotations were allowed. The dipole moment (μ_*BTB*_) of the planar *C*_3h_ symmetric molecule is, as expected, 0 D, whereas a net nearly perpendicular dipole moment of 2.3 D and 2.4 D for BTB was observed after allowing the carboxylic groups to rotate. If we define the extra work (*Wef*_*BTB*_) generated by the interaction of an electric field (*E*_*f*_) on BTB molecules as
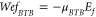
considering a bias potential range between 0.7 and 1.5 V and assuming an approximate distance of 1 nm between the tip and the sample, we calculated a *Wef*_*BTB*_ ranging from 0.3 to 0.56 eV (from 6.9 to 12.2 kcal/mol), comparable with the binding energy of BTB molecules on honeycomb structures at negative surfaces (approximately −10 kcal/mol, Figure 3). Thus, since both proton transfer and the rotation of COOH groups contribute to the loss of *C*_3h_ symmetry in the BTB molecules within the network, the generation of a dipole moment that aligns with the EEF induces the collapse of the structure.

## Conclusions

We have described the phase transition mechanism involved in supramolecular networks as a response to the EEF at the solid/liquid interface. By discussing each energetic contribution in a systematic fashion, we have proven that the switching effect is not inherent to any molecule but instead is related to a global energetic perspective of the adsorbate/substrate system under different experimental conditions. We have shown how a switchable molecule (such as BTB) becomes irresponsive to the EEF by increasing the adsorbate–substrate binding energy at the SLG/Cu/liquid interface. Additionally, we discussed the importance of the intermolecular H-bonding interactions on the switching mechanism by replacing -COOH with -CHO groups (BTB to C3-Ald). This functional group modification weakens the intermolecular H-bonds, preventing the expression of a porous CHO-based motif, and thus inhibits the switching from a stable close-packed structure to a potential unstable porous network. The effect of the surface polarity on the system’s stability was discussed based on DFT calculations performed on a graphene layer which determine that close-packed structures are more stable at positive sample bias, whereas at negative sample bias, all the structures lose overall stability. Finally, via reactive molecular dynamics studies, we have shown that the formation of polarizable species is correlated to the carboxyl group rotation and the dynamics of the proton exchange. This dynamic scenario is influenced by the applied electric field, promoting molecular switching to the most stable structures according to the surface polarity. The switching of supramolecular networks by an EEF is a fascinating effect, and here we analyze the factors that make a surface supported supramolecular network switchable, exploring the predictability and reversibility on the actuated interface.

## Methods and Experimental Section

Solutions of different concentrations of 1,3,5-tris(4-carboxyphenyl)benzene (BTB), trimesic acid (TMA) and 1,3,5-tris(4-formylphenyl)benzene (C3-Ald) were prepared in *n*-nonanoic acid (synthesis, Merck). Highly ordered pyrolytic graphite (HOPG, Bruker), ZYB grade, was used as substrate. HOPG substrate was cleaved with adhesive tape prior to use. Single layer graphene substrates deposited on Cu and on SiO_2_ were used as received from Graphenea without further cleaning. The samples were prepared by depositing 4 μL of the BTB solution on the HOPG substrate. STM tips were prepared by mechanical cutting of Pt/Ir wire (90%/10%, diameter 0.25 mm, GoodFellow). All STM measurements were performed by using a Bruker system at constant-current mode. After the samples were prepared, the tip was immersed in the droplet of solution at the liquid/solid interface. STM images were processed using WSXM 5.0 software.^36^

### Density Functional Theory Calculations

DFT calculations were performed with the Quantum Espresso (QE) package.^37^ The PBE formulation was used for the exchange and correlation functional^38^ together with norm-conserving ultrasoft pseudopotentials.^39^ The electron wave functions were expanded in a plane-wave basis set up to a kinetic energy cutoff of 40 Ry (240 Ry for the density). Due to the large cell sizes, only one k point (gamma) was used for integration in the first Brillouin to obtain a good balance between the number of atoms and the computational burden. Only in the case of graphene on copper, the integration in the first Brillouin zone was performed with a (2 × 2 × 1) Monkhorst–Pack mesh.^40^ Dispersive forces between BTB molecules and graphene were considered using Grimme’s semiempirical DFT-D2 approach^41^ as implemented in the PWscf code1 of QE. A vacuum thickness of 15 Å was introduced between the slabs.

### ReaxFF Molecular Dynamics Simulations

Reactive MD simulations were performed with the ReaxFF force field.^41,42^ We employed the ReaxFF force field reported by Sengul et al.^43^ MD simulations were performed with the ADF2020^44^ and LAMMPS^45^ codes. The temperature was controlled with a Berendsen thermostat^46^ (100 fs damping constant). The initial velocities were assigned according to the Boltzmann distribution. A Velocity-Verlet algorithm was used in the NVT/MD simulations with a 0.25 fs time step.

## Supporting Information Available

Details of the synthesis of the C3-Ald molecule and further STM images of BTB on SiO_2_ and of TMA on HOPG (PDF)
Sequential images generated by molecular dynamics simulations showing that polarizable species created by the H-exchange process and carboxylic group rotations can induce molecular switching (MP4)

## Abbreviations

HOPG: highly ordered pyrolytic graphite
BTB: 1,3,5-tris(4-carboxyphenyl)benzene
SAMs: self-assembled monolayers
STM: scanning tunneling microscope
NA: *n*-nonanoic acid
HA: *n*-heptanoic acid
C_0_: critical concentration
DFT: density functional theory
P: intrinsic dipole moment
TMA: trimesic acid
UHV: ultrahigh vacuum

## References

[ref1] Barth J. V.; Costantini G.; Kern K. Engineering Atomic and Molecular Nanostructures at Surfaces. Nature 2005, 437 (7059), 671–679. 10.1038/nature04166. 16193042

[ref2] Kudernac T.; Lei S.; Elemans J. A. A. W.; De Feyter S. Two-Dimensional Supramolecular Self-Assembly: Nanoporous Networks on Surfaces. Chem. Soc. Rev. 2009, 38 (2), 402–421. 10.1039/B708902N. 19169457

[ref3] Goronzy D. P.; Ebrahimi M.; Rosei F.; Arramel; Fang Y.; De Feyter S.; Tait S. L.; Wang C.; Beton P. H.; Wee A. T. S.; Weiss P. S.; Perepichka D. F. Supramolecular Assemblies on Surfaces: Nanopatterning, Functionality, and Reactivity. ACS Nano 2018, 12 (8), 7445–7481. 10.1021/acsnano.8b03513. 30010321

[ref4] Elemans J. A. A. W. Externally Applied Manipulation of Molecular Assemblies at Solid-Liquid Interfaces Revealed by Scanning Tunneling Microscopy. Adv. Funct. Mater. 2016, 26 (48), 8932–8951. 10.1002/adfm.201603145.

[ref5] Casalini S.; Bortolotti C. A.; Leonardi F.; Biscarini F. Self-Assembled Monolayers in Organic Electronics. Chem. Soc. Rev. 2017, 46 (1), 40–71. 10.1039/C6CS00509H. 27722675

[ref6] Gutzler R.; Stepanow S.; Grumelli D.; Lingenfelder M.; Kern K. Mimicking Enzymatic Active Sites on Surfaces for Energy Conversion Chemistry. Acc. Chem. Res. 2015, 48 (7), 2132–2139. 10.1021/acs.accounts.5b00172. 26121410

[ref7] Teyssandier J.; Feyter S. D.; Mali K. S. Host–Guest Chemistry in Two-Dimensional Supramolecular Networks. Chem. Commun. 2016, 52 (77), 11465–11487. 10.1039/C6CC05256H. 27709179

[ref8] Lee S.-L.; Fang Y.; Velpula G.; Cometto F. P.; Lingenfelder M.; Müllen K.; Mali K. S.; De Feyter S. Reversible Local and Global Switching in Multicomponent Supramolecular Networks: Controlled Guest Release and Capture at the Solution/Solid Interface. ACS Nano 2015, 9 (12), 11608–11617. 10.1021/acsnano.5b06081. 26550765

[ref9] Iritani K.; Tahara K.; De Feyter S.; Tobe Y. Host–Guest Chemistry in Integrated Porous Space Formed by Molecular Self-Assembly at Liquid–Solid Interfaces. Langmuir 2017, 33 (19), 4601–4618. 10.1021/acs.langmuir.7b00083. 28206764

[ref10] Cui D.; Liu C.-H.; Rosei F.; Perepichka D. F. Bidirectional Phase Transformation of Supramolecular Networks Using Two Molecular Signals. ACS Nano 2022, 16 (1), 1560–1566. 10.1021/acsnano.1c10122. 35014801

[ref11] Lackinger M.; Heckl W. M. Carboxylic Acids: Versatile Building Blocks and Mediators for Two-Dimensional Supramolecular Self-Assembly. Langmuir 2009, 25 (19), 11307–11321. 10.1021/la900785f. 19453128

[ref12] MacLeod J. Design and Construction of On-Surface Molecular Nanoarchitectures: Lessons and Trends from Trimesic Acid and Other Small Carboxlyated Building Blocks. J. Phys. Appl. Phys. 2020, 53 (4), 043002 10.1088/1361-6463/ab4c4d.

[ref13] Cometto F. P.; Kern K.; Lingenfelder M. Local Conformational Switching of Supramolecular Networks at the Solid/Liquid Interface. ACS Nano 2015, 9 (5), 5544–5550. 10.1021/acsnano.5b01658. 25857528

[ref14] Thi Ngoc Ha N.; Gopakumar T. G.; Hietschold M. Polymorphism Driven by Concentration at the Solid–Liquid Interface. J. Phys. Chem. C 2011, 115 (44), 21743–21749. 10.1021/jp111640t.

[ref15] Meier C.; Roos M.; Künzel D.; Breitruck A.; Hoster H. E.; Landfester K.; Gross A.; Behm R. J.; Ziener U. Concentration and Coverage Dependent Adlayer Structures: From Two-Dimensional Networks to Rotation in a Bearing. J. Phys. Chem. C 2010, 114 (2), 1268–1277. 10.1021/jp910029z.

[ref16] Ochs O.; Hocke M.; Spitzer S.; Heckl W. M.; Martsinovich N.; Lackinger M. Origin of Solvent-Induced Polymorphism in Self-Assembly of Trimesic Acid Monolayers at Solid–Liquid Interfaces. Chem. Mater. 2020, 32 (12), 5057–5065. 10.1021/acs.chemmater.0c00827.

[ref17] Yang Y.; Wang C. Solvent Effects on Two-Dimensional Molecular Self-Assemblies Investigated by Using Scanning Tunneling Microscopy. Curr. Opin. Colloid Interface Sci. 2009, 14 (2), 135–147. 10.1016/j.cocis.2008.10.002.

[ref18] Ha N. T. N.; Gopakumar T. G.; Hietschold M. Polymorphs of Trimesic Acid Controlled by Solvent Polarity and Concentration of Solute at Solid–Liquid Interface. Surf. Sci. 2013, 607, 68–73. 10.1016/j.susc.2012.08.008.

[ref19] Kampschulte L.; Lackinger M.; Maier A.-K.; Kishore R. S. K.; Griessl S.; Schmittel M.; Heckl W. M. Solvent Induced Polymorphism in Supramolecular 1,3,5-Benzenetribenzoic Acid Monolayers. J. Phys. Chem. B 2006, 110 (22), 10829–10836. 10.1021/jp057553m. 16771333

[ref20] Ubink J.; Enache M.; Stöhr M. Bias-Induced Conformational Switching of Supramolecular Networks of Trimesic Acid at the Solid-Liquid Interface. J. Chem. Phys. 2018, 148 (17), 174703 10.1063/1.5017930. 29739202

[ref21] Lackinger M.; Griessl S.; Heckl W. M.; Hietschold M.; Flynn G. W. Self-Assembly of Trimesic Acid at the Liquid–Solid Interfacea Study of Solvent-Induced Polymorphism. Langmuir 2005, 21 (11), 4984–4988. 10.1021/la0467640. 15896040

[ref22] Nguyen D. C. Y.; Smykalla L.; Nguyen T. N. H.; Rüffer T.; Hietschold M. Deposition-Temperature- and Solvent-Dependent 2D Supramolecular Assemblies of Trimesic Acid at the Liquid–Graphite Interface Revealed by Scanning Tunneling Microscopy. J. Phys. Chem. C 2016, 120 (20), 11027–11036. 10.1021/acs.jpcc.6b03409.

[ref23] Mahmood A.; Zeng X.; Saleemi A. S.; Cheng K.-Y.; Lee S.-L. Electric-Field-Induced Supramolecular Phase Transitions at the Liquid/Solid Interface: Cat-Assembly from Solvent Additives. Chem. Commun. 2020, 56 (62), 8790–8793. 10.1039/D0CC01670E. 32618318

[ref24] Gutzler R.; Sirtl T.; Dienstmaier J. F.; Mahata K.; Heckl W. M.; Schmittel M.; Lackinger M. Reversible Phase Transitions in Self-Assembled Monolayers at the Liquid-Solid Interface: Temperature-Controlled Opening and Closing of Nanopores. J. Am. Chem. Soc. 2010, 132 (14), 5084–5090. 10.1021/ja908919r. 20235537

[ref25] Ruben M.; Payer D.; Landa A.; Comisso A.; Gattinoni C.; Lin N.; Collin J.-P.; Sauvage J.-P.; De Vita A.; Kern K. 2D Supramolecular Assemblies of Benzene-1,3,5-Triyl-Tribenzoic Acid: Temperature-Induced Phase Transformations and Hierarchical Organization with Macrocyclic Molecules. J. Am. Chem. Soc. 2006, 128 (49), 15644–15651. 10.1021/ja063601k. 17147373

[ref26] Cometto F.; Frank K.; Stel B.; Arisnabarreta N.; Kern K.; Lingenfelder M. The STM Bias Voltage-Dependent Polymorphism of a Binary Supramolecular Network. Chem. Commun. 2017, 53 (83), 11430–11432. 10.1039/C7CC06597C. 28975932

[ref27] Velpula G.; Teyssandier J.; De Feyter S.; Mali K. S. Nanoscale Control over the Mixing Behavior of Surface-Confined Bicomponent Supramolecular Networks Using an Oriented External Electric Field. ACS Nano 2017, 11 (11), 10903–10913. 10.1021/acsnano.7b04610. 29112378 PMC5707626

[ref28] Saeed M.; Mahmood A.; Saleemi A. S.; Zeng X.; Lee S.-L. Supramolecular Self-Assembly: Molecular Polymorphs and Their Transitions Triggered Electrically via Water Assistance at the Liquid/Graphite Interface. J. Phys. Chem. C 2020, 124 (1), 829–835. 10.1021/acs.jpcc.9b11006.

[ref29] Mahmood A.; Saeed M.; Chan Y.; Saleemi A. S.; Guo J.; Lee S.-L. Synergic Effect: Temperature-Assisted Electric-Field-Induced Supramolecular Phase Transitions at the Liquid/Solid Interface. Langmuir 2019, 35 (24), 8031–8037. 10.1021/acs.langmuir.9b00569. 31120252

[ref30] Chan Y.; Khan S. B.; Mahmood A.; Saleemi A. S.; Lian Z.; Ren Y.; Zeng X.; Lee S.-L. Electrical-Pulse-Induced Mixture and Separation in Surface Supramolecular Hybrids: STM Experiments and Theoretical Approaches. J. Phys. Chem. C 2020, 124 (1), 815–821. 10.1021/acs.jpcc.9b10537.

[ref31] Khomyakov P. A.; Giovannetti G.; Rusu P. C.; Brocks G.; van den Brink J.; Kelly P. J. First-Principles Study of the Interaction and Charge Transfer between Graphene and Metals. Phys. Rev. B 2009, 79 (19), 195425 10.1103/PhysRevB.79.195425.

[ref32] Ibenskas A.; Tornau E. E. Modeling of Ribbon and Oblique Structures of Benzene-1,3,5-Triyl-Tribenzoic Acid. J. Phys. Chem. C 2020, 124 (34), 18650–18659. 10.1021/acs.jpcc.0c05790.

[ref33] Payer D.; Comisso A.; Dmitriev A.; Strunskus T.; Lin N.; Wöll C.; DeVita A.; Barth J. V.; Kern K. Ionic Hydrogen Bonds Controlling Two-Dimensional Supramolecular Systems at a Metal Surface. Chem. – Eur. J. 2007, 13 (14), 3900–3906. 10.1002/chem.200601325. 17290466

[ref34] Li J.; Gottardi S.; Solianyk L.; Moreno-López J. C.; Stöhr M. 1,3,5-Benzenetribenzoic Acid on Cu(111) and Graphene/Cu(111): A Comparative STM Study. J. Phys. Chem. C 2016, 120 (32), 18093–18098. 10.1021/acs.jpcc.6b05541. PMC5002934 27588158

[ref35] Henkelman G.; Uberuaga B. P.; Jónsson H. A Climbing Image Nudged Elastic Band Method for Finding Saddle Points and Minimum Energy Paths. J. Chem. Phys. 2000, 113 (22), 9901–9904. 10.1063/1.1329672.

[ref36] Horcas I.; Fernández R.; Gómez-Rodríguez J. M.; Colchero J.; Gómez-Herrero J.; Baro A. M. WSXM: A Software for Scanning Probe Microscopy and a Tool for Nanotechnology. Rev. Sci. Instrum. 2007, 78 (1), 013705 10.1063/1.2432410. 17503926

[ref37] Giannozzi P.; Baseggio O.; Bonfà P.; Brunato D.; Car R.; Carnimeo I.; Cavazzoni C.; de Gironcoli S.; Delugas P.; Ferrari Ruffino F.; Ferretti A.; Marzari N.; Timrov I.; Urru A.; Baroni S. QUANTUM ESPRESSO toward the Exascale. J. Chem. Phys. 2020, 152 (15), 154105 10.1063/5.0005082. 32321275

[ref38] Perdew J. P.; Burke K.; Ernzerhof M. Generalized Gradient Approximation Made Simple. Phys. Rev. Lett. 1996, 77 (18), 3865–3868. 10.1103/PhysRevLett.77.3865. 10062328

[ref39] Vanderbilt D. Soft Self-Consistent Pseudopotentials in a Generalized Eigenvalue Formalism. Phys. Rev. B 1990, 41 (11), 7892–7895. 10.1103/PhysRevB.41.7892. 9993096

[ref40] Monkhorst H. J.; Pack J. D. Special Points for Brillouin-Zone Integrations. Phys. Rev. B 1976, 13 (12), 5188–5192. 10.1103/PhysRevB.13.5188.

[ref41] Grimme S. Semiempirical GGA-Type Density Functional Constructed with a Long-Range Dispersion Correction. J. Comput. Chem. 2006, 27 (15), 1787–1799. 10.1002/jcc.20495. 16955487

[ref42] van Duin A. C. T.; Dasgupta S.; Lorant F.; Goddard W. A. ReaxFF: A Reactive Force Field for Hydrocarbons. J. Phys. Chem. A 2001, 105 (41), 9396–9409. 10.1021/jp004368u.

[ref43] Sengul M. Y.; Randall C. A.; van Duin A. C. T. ReaxFF Molecular Dynamics Simulation of Intermolecular Structure Formation in Acetic Acid-Water Mixtures at Elevated Temperatures and Pressures. J. Chem. Phys. 2018, 148 (16), 164506 10.1063/1.5025932. 29716228

[ref44] te Velde G.; Bickelhaupt F. M.; Baerends E. J.; Fonseca Guerra C.; van Gisbergen S. J. A.; Snijders J. G.; Ziegler T. Chemistry with ADF. J. Comput. Chem. 2001, 22 (9), 931–967. 10.1002/jcc.1056.

[ref45] Plimpton S. Fast Parallel Algorithms for Short-Range Molecular Dynamics. J. Comput. Phys. 1995, 117 (1), 1–19. 10.1006/jcph.1995.1039.

[ref46] Berendsen H. J. C.; Postma J. P. M.; van Gunsteren W. F.; DiNola A.; Haak J. R. Molecular Dynamics with Coupling to an External Bath. J. Chem. Phys. 1984, 81, 3684–3690. 10.1063/1.448118.

