# Supplementary material for: Rational Design of 2D Supramolecular Networks Switchable by External Electric Fields
Source: ACS Nano. 2024 Jan 23;18(5):4287–96. doi: 10.1021/acsnano.3c09775 (PMC10851663; doi:10.1021/acsnano.3c09775)
Supplement: Supplementary file 1 — nn3c09775_si_001.pdf [file nn3c09775_si_001.pdf]

Supporting Information

Rational Design of 2D Supramolecular Networks Switchable by External Electric Fields.

Fernando P. Cometto,[1,2,3] Nicolás Arisnabarreta, [1,2,3] Radovan Vanta,[1] Daniela K. Jacquelín,[2] Vijay Vyas,[4] Bettina V. Lotsch,[4,5] Patricia A. Paredes-Olivera,[6] E. Martín Patrito[2,3] and Magalí Lingenfelder[1*]*

1. Max Planck-EPFL Laboratory for Molecular Nanoscience and IPHYS, EPFL, Lausanne, CH 1015, Switzerland.

2. Instituto de Investigaciones en Fisicoquímica de Córdoba (INFIQC), CONICET, Ciudad Universitaria, Córdoba, X5000HUA, Argentina.

3. Departamento de Fisicoquímica, Facultad de Ciencias Químicas, Universidad Nacional de Córdoba (UNC), Ciudad Universitaria, Córdoba, X5000HUA, Argentina.

4. Max Planck Institute for Solid State Research, Stuttgart, D-70569, Germany.

5. Department of Chemistry, University of Munich (LMU), 81377 Munich, Germany.

6. Departamento de Química Teórica y Computacional, Facultad de Ciencias Químicas, Universidad Nacional de Córdoba (UNC), Ciudad Universitaria, Córdoba, X5000HUA, Argentina.

Synthesis of 1,3,5-Tris(4-formylphenyl)benzene (C3-Ald)

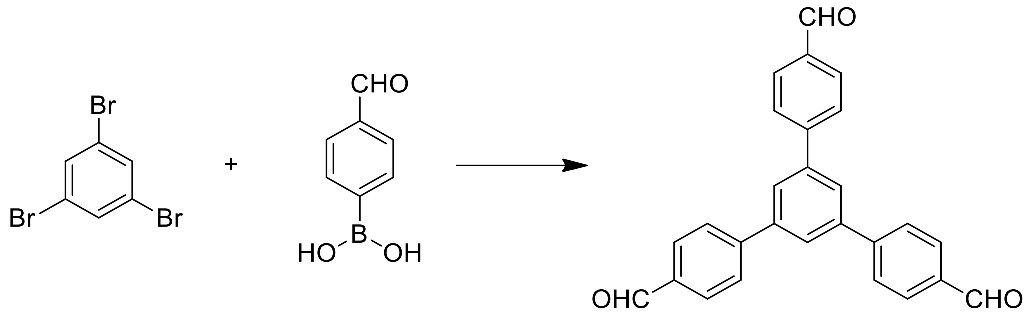

This compound was prepared as per our earlier reported procedure.[1] In a Schlenk flask equipped with a stir bar and reflux condenser, 1, 3, 5-tribromobenzene (200 mg, 0.62 mmol), 4-formylphenyl boronic acid (0.44 g, 2.8 mmol), potassium carbonate (0.42 g, 3.0 mmol), and palladiumtetrakis(triphenylphosphine) (35 mg, 0.03 mmol) were taken. The flask was evacuated and backfilled with argon three times. To this were added toluene (25 mL), ethanol (5 mL) and water (5 mL) and the contents were refluxed for 48 h. The reaction mixture was then cooled to room temperature and water (50 mL) was added. The organic compound was extracted with dichloromethane, dried over anhydrous $MgSO_4$, and then filtered. The solvent was removed under reduced pressure and after column chromatography (silica gel, dichloromethane) pure product was obtained as an off white solid (0.18 g, 74 %). ^1H NMR ($CDCl_3$, 300 MHz): δ ppm 10.11 (s, 3H), 8.02 (d, J = 8.3 Hz, 6H), 7.91 (s, 3H), 7.87 (d, J = 8.3 Hz, 6H). ^{13}C NMR ($CDCl_3$, 75 MHz):

δ ppm 191.93, 146.49, 141.81, 135.99, 130.64, 128.19, 126.68. MALDI-TOF-MS for $C_{27}H_{18}O_3$: 389.8 M^+ (calculated: 390.1).

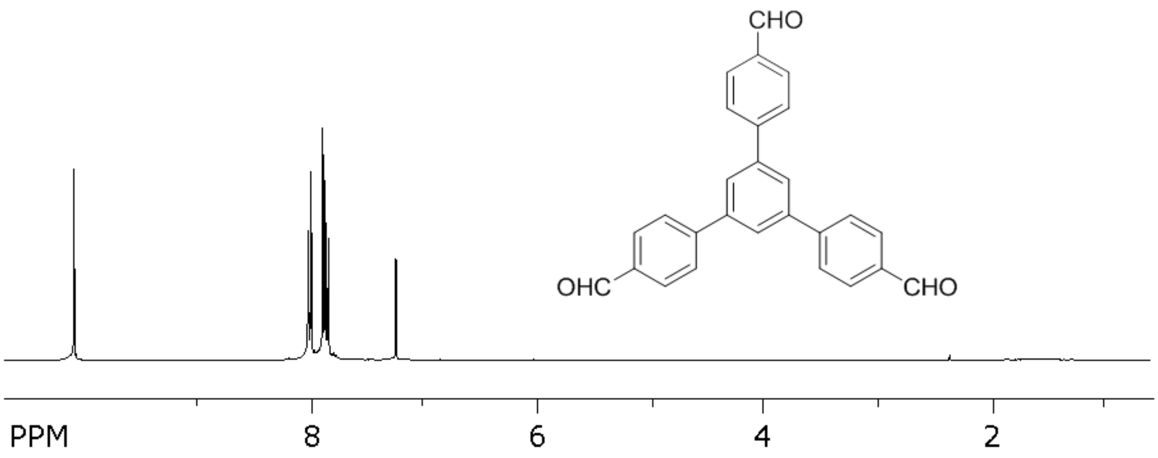

^1H NMR of 1,3,5-Tris(4-formylphenyl)benzene (**C3-Ald**).

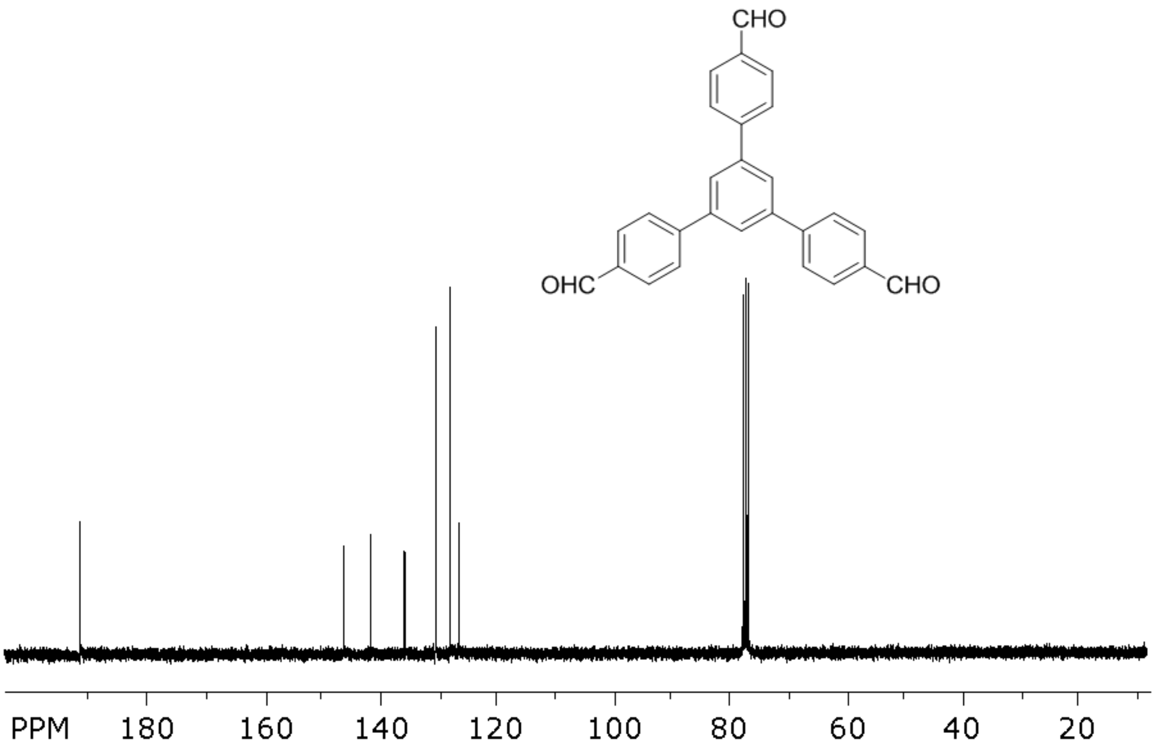

^{13}C NMR of 1,3,5-Tris(4-formylphenyl)benzene (**C3-Ald**).

1. Nat. Commun. 6:8508 doi: 10.1038/ncomms9508 (2015).

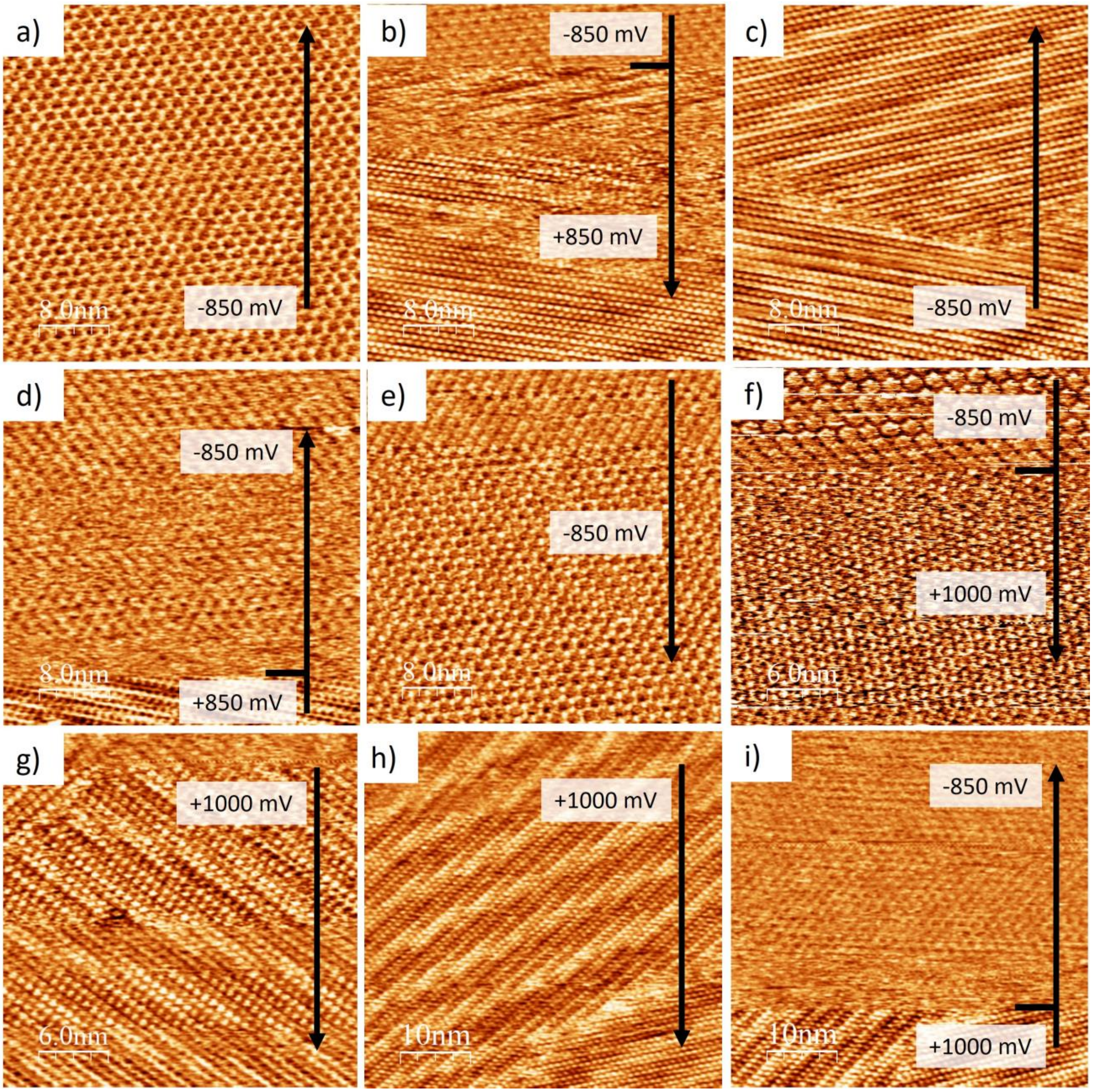

Figure S1. Consecutive STM images showing the switching behaviour of TMA on HOPG at the solid/liquid interface obtained at different bias voltages (V_{bias} = from +1000 to -850 mV; I = 150 pA).

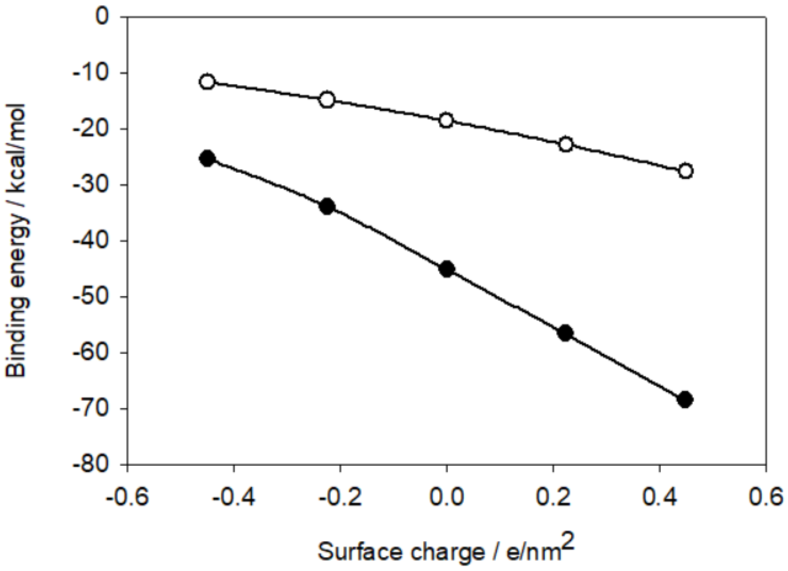

Figure S2 – Binding energy of a single BTB molecule (full circles) or a single nonanoic acid (empty circles) on grapheme *vs* the surface charge obtained by DFT calculations.

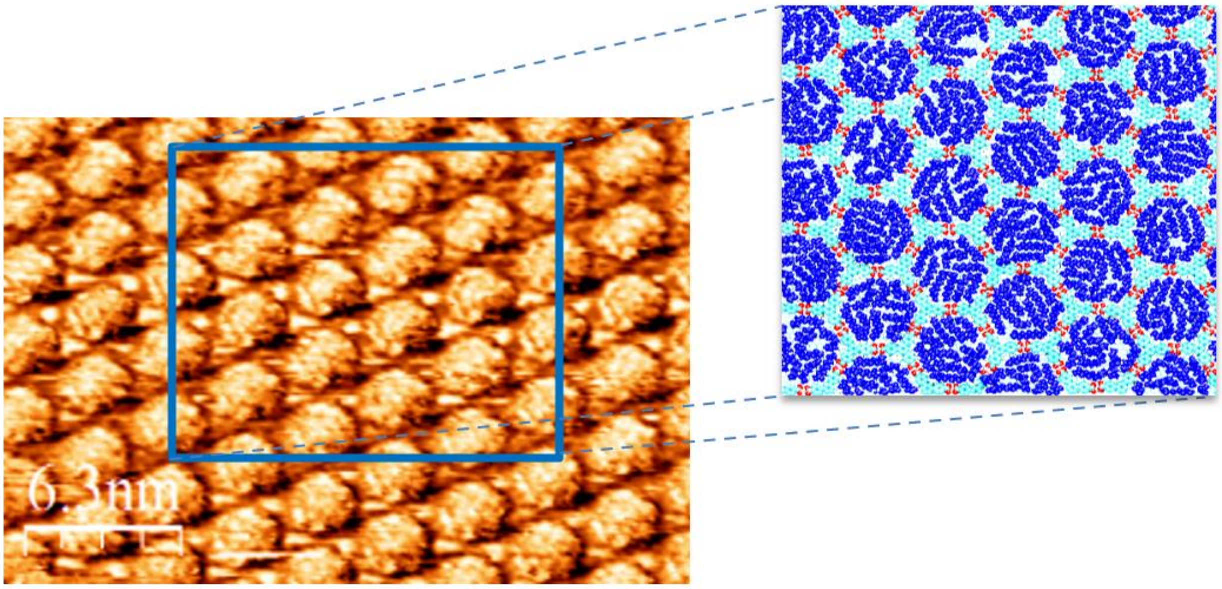

Figure S3 – STM image that shows BTB molecules and solvent molecules inside the pores. According to reactive molecular dynamics calculations up to 10 nonanoic solvent molecules can be accommodated inside the pore in the honeycomb BTB structure.

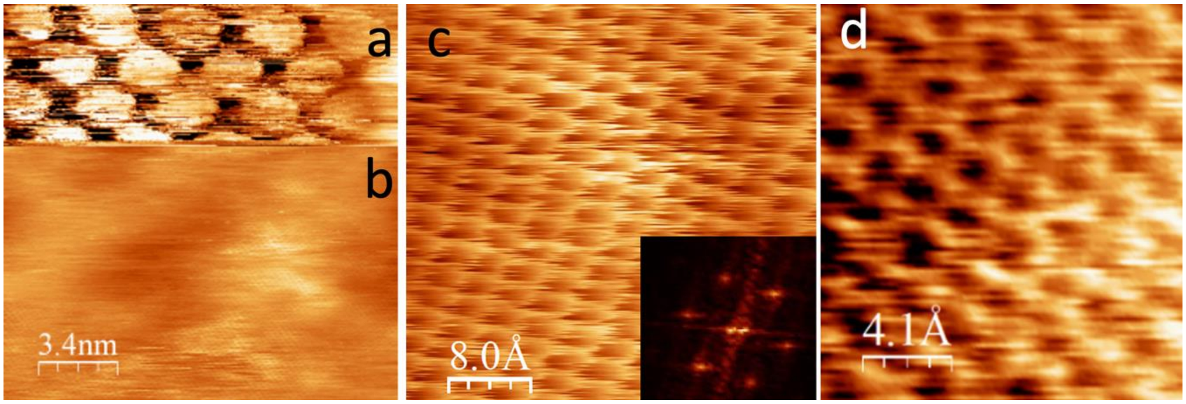

Figure S4: STM image showing the coexistence of BTB on the conserved graphene lattice in the BTB/SLG/SiO2 system ((a) V= -850 mV, molecules seen at 17.4 pA and (b, c) HOPG seen at 300 pA, inset in (c) FTT of the STM image showing the hexagonal graphene lattice). (d) High-resolution STM image showing graphene lattice in the BTB/SLG/Cu system (0.018 mV, 250 pA).

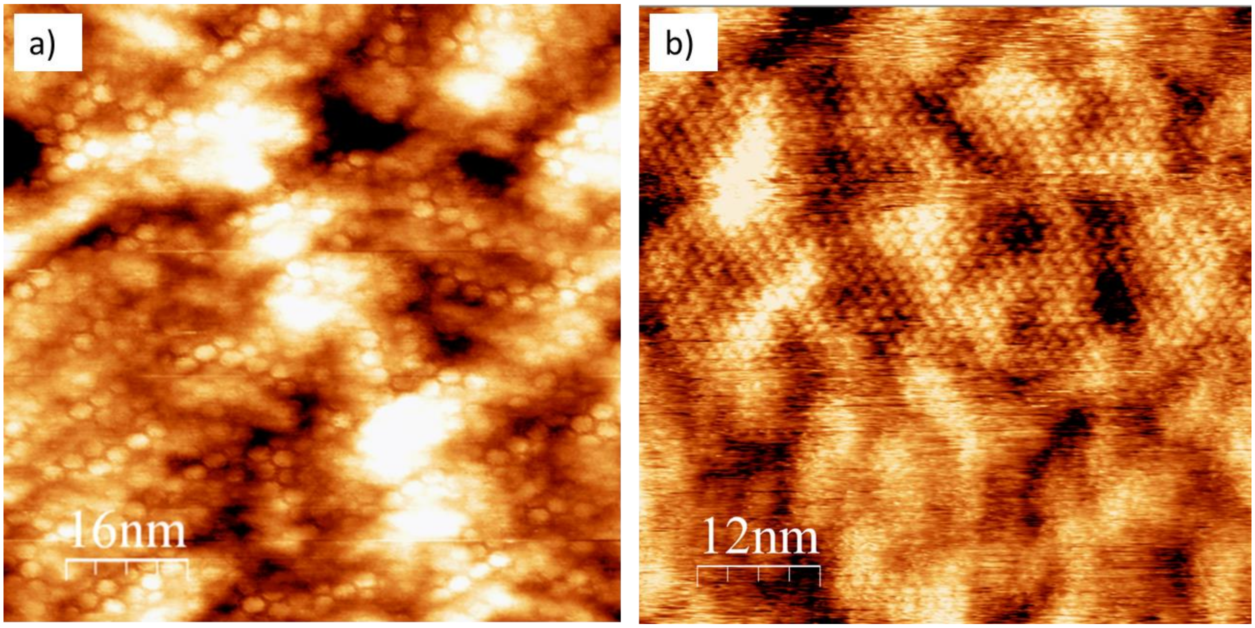

Figure S5 – STM images of BTB on SLG/SiO2 at different sample biases: a) at -850 mV and b) at +950 mV (I = 67 pA).

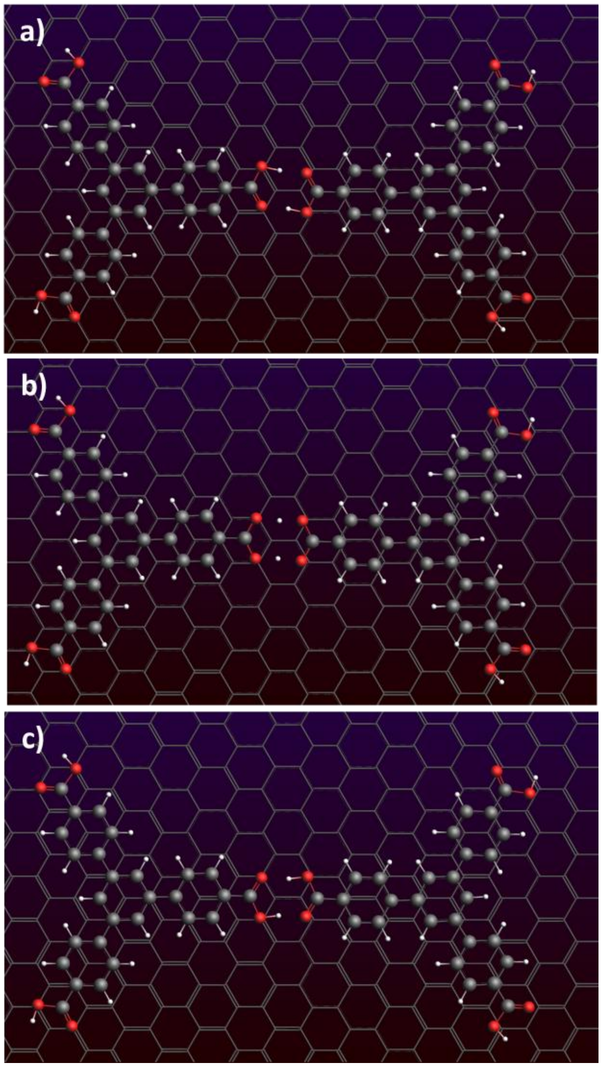

Figure S6 – Nudge elastic band calculation to obtain the energy barrier for proton exchange (0.47 eV). The Figures a, b and c show the initial, transition and final states for proton exchange between adjacent carboxylic groups of two BTB molecules adsorbed on graphene, respectively.
